# *In Vivo* Outer Hair Cell Length Changes Expose the Active Process in the Cochlea

**DOI:** 10.1371/journal.pone.0032757

**Published:** 2012-04-09

**Authors:** Dingjun Zha, Fangyi Chen, Sripriya Ramamoorthy, Anders Fridberger, Niloy Choudhury, Steven L. Jacques, Ruikang K. Wang, Alfred L. Nuttall

**Affiliations:** 1 Oregon Hearing Research Center, Oregon Health and Science University, Portland, Oregon, United States of America; 2 Department of Otolaryngology/Head and Neck Surgery, Xijing Hospital, Fourth Military Medical University, Shaanxi, People's Republic of China; 3 Department of Biomedical Engineering, Oregon Health and Science University, Portland, Oregon, United States of America; 4 Department of Dermatology, Oregon Health and Science University, Portland, Oregon, United States of America; 5 Department of Bioengineering, University of Washington, Seattle, Washington, United States of America; 6 Karolinska Institutet, Center for Hearing and Communication Research, Department of Clinical Science, Intervention, and Technology, M1 Karolinska University Hospital, Stockholm, Sweden; 7 Department of Biomedical Engineering, Michigan Technological University, Houghton, Michigan, United States of America; 8 Kresge Hearing Research Institute, The University of Michigan, Ann Arbor, Michigan, United States of America; Claremont Colleges, United States of America

## Abstract

**Background:**

Mammalian hearing is refined by amplification of the sound-evoked vibration of the cochlear partition. This amplification is at least partly due to forces produced by protein motors residing in the cylindrical body of the outer hair cell. To transmit power to the cochlear partition, it is required that the outer hair cells dynamically change their length, in addition to generating force. These length changes, which have not previously been measured *in vivo*, must be correctly timed with the acoustic stimulus to produce amplification.

**Methodology/Principal Findings:**

Using *in vivo* optical coherence tomography, we demonstrate that outer hair cells in living guinea pigs have length changes with unexpected timing and magnitudes that depend on the stimulus level in the sensitive cochlea.

**Conclusions/Significance:**

The level-dependent length change is a necessary condition for directly validating that power is expended by the active process presumed to underlie normal hearing.

## Introduction

The remarkable sensitivity and frequency resolution of mammalian hearing organs [1], [2], [3], [4], [5] stems from the fast motility of outer hair cells (OHCs) [6], [7], [8]. When the cochlea is functioning normally, this motility may boost basilar membrane (BM) vibrations by several tens of decibels [9], [10], by adding energy into the sound-evoked motion.

The central element of this process are the OHCs, which are capable of electrical-to-mechanical transduction, e.g. alterations in their membrane potential, induced by the gating of mechanically sensitive ion channels, leading to changes in cell length [6], [11]. This process has been observed *in vitro* in isolated OHCs [6], [12] and *in situ* in the low-frequency regions of cochlear explants [7] and recently published measurements suggest that length changes also occur *in vivo* [13].To be effective in augmenting sound-evoked vibrations *in vivo*, the length changes must be correctly synchronized to the acoustic stimulus. The most widely held hypothesis, as stated by Gummer et al. [14], suggests that amplification occurs if maximal OHC contraction coincides with maximal BM velocity in the direction of scala vestibuli. Other timing relationships are thought to cause smaller amplification or even attenuation.

Indirect experimental evidence for OHC electromotility *in vivo* comes from the electrically driven salicylate-sensitive BM velocity measured up to 100 kHz in guinea pigs [15]. However, direct characterization for the OHC length change *in vivo* is still lacking. Apart from their length change, isolated OHCs also demonstrate a voltage-dependent change in stiffness [16]. If the OHC stiffness change *in vivo* were larger than what is demonstrated in the isolated cells, such a phasic modulation could lead to amplified sound-evoked movements in the absence of large length changes. To understand how the vibration of the cochlear partition is boosted *in vivo*, it is therefore important to determine amplitude and timing of OHC length changes relative to the sound stimulus. In this report, we focus on the measurement and direct characterization of the OHC length-change *in vivo* over a wide range of stimulus levels.

OHCs occupy a mechanically privileged place in the inner ear, connecting the reticular lamina (RL) with the BM through the body of the Deiters' cells [17], [18]. In this connection, the OHC is the only cell with fast motility and capacity for significant change of cell length [19], [20], [21]. Thus, should OHCs have sufficient strength to act on the impedances of the organ of Corti complex, they will move the RL and the BM independently *in vivo*.

To directly measure OHCs length changes, we used an optical coherence tomography system [13], [22], [23] that allows imaging and vibration measurements from both the BM and the RL in living anaesthetized guinea pigs. We show that OHCs length changes indeed exist in the sensitive cochlea, that these length changes depend on the sound stimulus level, and that the polarity of the length change differs from accepted theory. These salient features of OHCs *in vivo* provide experimental evidence for mechanical power generation by OHCs and show that the current models of hearing organ function may need to be revised.

## Results

### Data collection and consistency of the data

**Figure 1:**
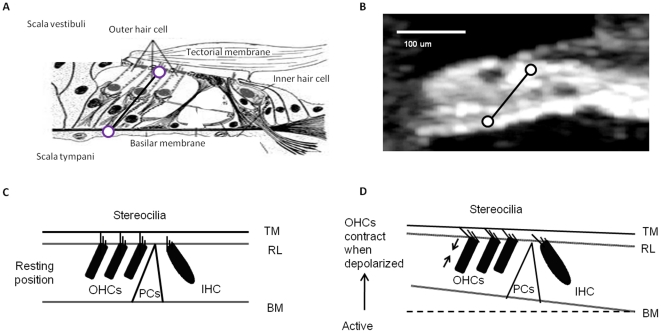
The cochlea structure and function. **a**. The illustrated organ of Corti in cross-section. **b**. An OCT image of the organ of Corti *in vivo*. Circles mark the locations of vibration measurement. **c**. A cartoon of hair cell excitation without OHC length change. **d**. A cartoon showing that when depolarized, OHCs contract to become shorter in length. This will draw together the reticular lamina and basilar membrane; IHC, inner hair cell; OHC, outer hair cell; PCs, pillar cells.

In this study of the dynamic length of OHCs, we first obtained an organ of Corti image with recognizable cellular morphology using the imaging mode of the optical coherence tomography system. From such images, the angle between the cell body of the OHCs and the optical axis can be determined (**Fig. 1a, b**, see Methods), which made it possible to determine the appropriate measurement locations for the RL and BM. Thirty-three animals were used in this study, but of the 33, 17 were excluded because surgical trauma caused more than 15 dB loss of auditory sensitivity. Incomplete data were collected from an additional 10 cochleae, where the auditory sensitivity declined during data acquisition, leaving six preparations, where a complete data set was acquired. The present report is based on data from these six preparations.

### Difference of sound-induced vibration of the BM and RL

**Figure 2:**
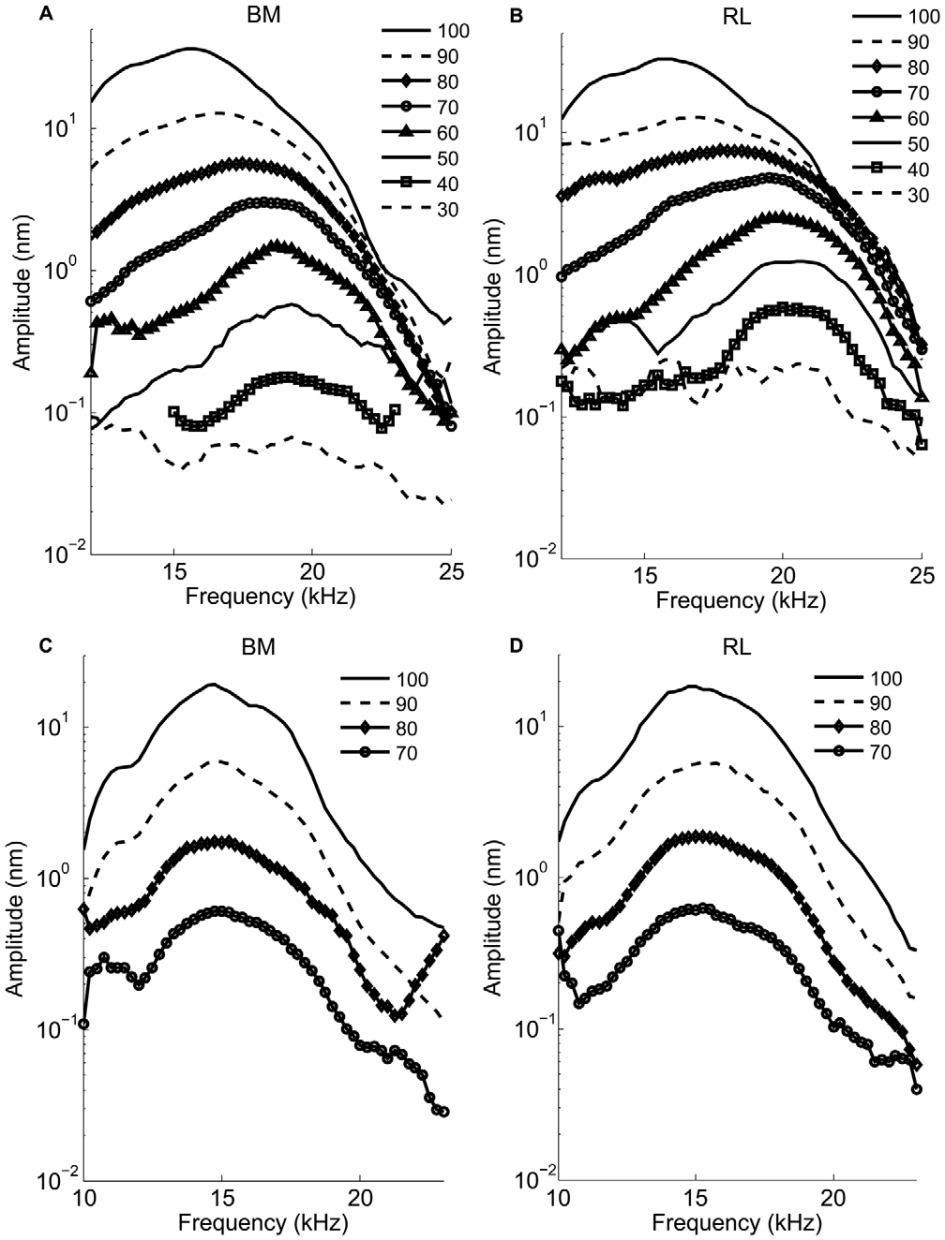
Vibration magnitude spectra of the basilar membrane (BM, -panels a, c) and reticular lamina (RL, -panels b, d) in a sensitive guinea pig cochlea and a postmortem guinea pig cochlea. Displacement magnitudes of vibration in nm are plotted as a function of stimulus frequency with sound level as the parameter. For these data, an auditory sensitivity loss of 12 dB is caused by the surgical preparation. Numbers against each curve represent sound levels of dB SPL at the eardrum used to stimulate the hearing organ vibration. In living animals (**a, b**), at 30 dB SPL, the maximum amplitude of BM vibration is 0.05 nm, the maximum displacement amplitude RL of is 0.1 nm at the best frequency of 20 kHz. In the postmortem animal(**c, d**), at 70 dB SPL, the maximum amplitude of BM vibration is 0.52 nm, the maximum displacement amplitude of the RL is 0.51 nm.

In the sensitive cochlea, BM vibrations are highly frequency-tuned (**Fig. 2a**). At low levels of stimulation, a distinct peak, near 20 kHz in this example, is flanked by rapidly decaying segments on either side. As the stimulus level increases, the peak moves toward lower frequencies, but the response amplitude does not grow in proportion to the increase of the stimulus level. This nonlinearity characterizes the sensitive cochlea and has been described in many previous studies [3], [4], [24].

**Figure 3:**
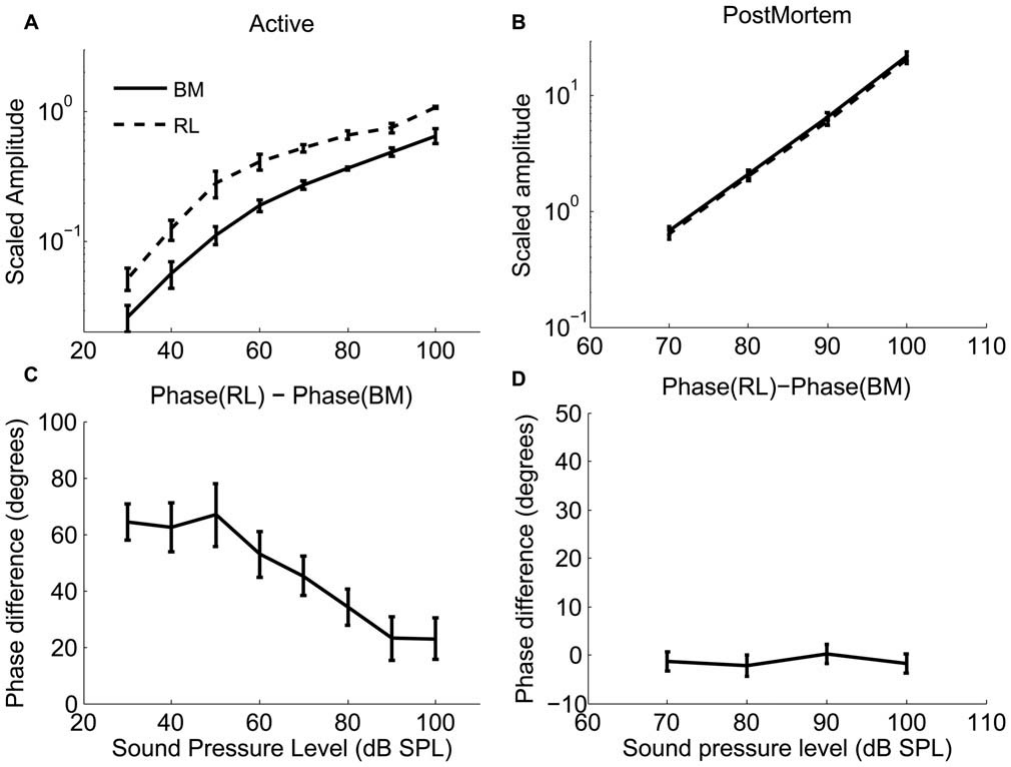
Displacement magnitude difference (a, b) and the phase difference(c, d) as a function of the sound level between the basilar membrane (BM) and reticular lamina (RL). The mean ± standard error of the BM vibration magnitude (solid lines) and the RL displacement (dashed lines) and the displacement difference between the RL and the BM as a function of sound level from live animals (**a**) and postmortem animals (**b**); The phase difference between the RL and the BM magnitude and the phase as a function of the sound level from live animals (**c**) and postmortem animals (**d**). In order to group the animals, the frequencies were normalized with respect to the best frequency, which varied among the animals at the chosen measurement locations. This compensates for the slight variations in the best frequencies in the different experiments (best frequency range, 18.75–22.25 kHz). BF_P_ is around 15 kHz at postmortem in the experiments.

At the RL, displacements are also highly tuned, but they have a greater magnitude than those of the BM. At 30 dB SPL, peak RL displacements are a factor two larger than those of the BM, a difference that becomes smaller as the stimulus level increases (**Fig. 2b**). This difference in magnitude is only seen in sensitive ears and disappears following the death of the animal (cf. **Fig. 2c** and **d**). Note that the level-dependent shift in the position of the peak and the nonlinear increase of the response magnitude are absent postmortem (**Fig. 2c** and **d**). To illustrate these differences, we plot displacement magnitudes as a function of stimulus intensity in **Fig. 3a** (mean and standard errors from the six sensitive preparations). At 30 dB SPL and at the best frequency, RL vibrations are a factor 2.2±0.3 larger than the BM (n = 6, p?0.05). When the stimulus level increases, the difference becomes smaller, but it remains statistically significant at 100 dB SPL (1.8±0.3, p<0.05). The two structures also show a pronounced level-dependent alteration in response phase. At low levels, RL vibrations phase-lead the BM, but this difference in response timing becomes less pronounced as the stimulus intensity increases (**Fig. 3c**). Postmortem, vibrations are much smaller, grow linearly with stimulus intensity, and different structures within the organ of Corti have nearly identical vibration amplitudes and phases (**Fig. 3b** and **d**, n = 4). Given the cochlear sensitivity to trauma and the well-known disappearance of the active mechanism following death, the most parsimonious explanation of these data is that motility of the OHCs is the primary cause of the displacement difference between RL and BM.

### Frequency dependence and nonlinearity of OHC length change

**Figure 4:**
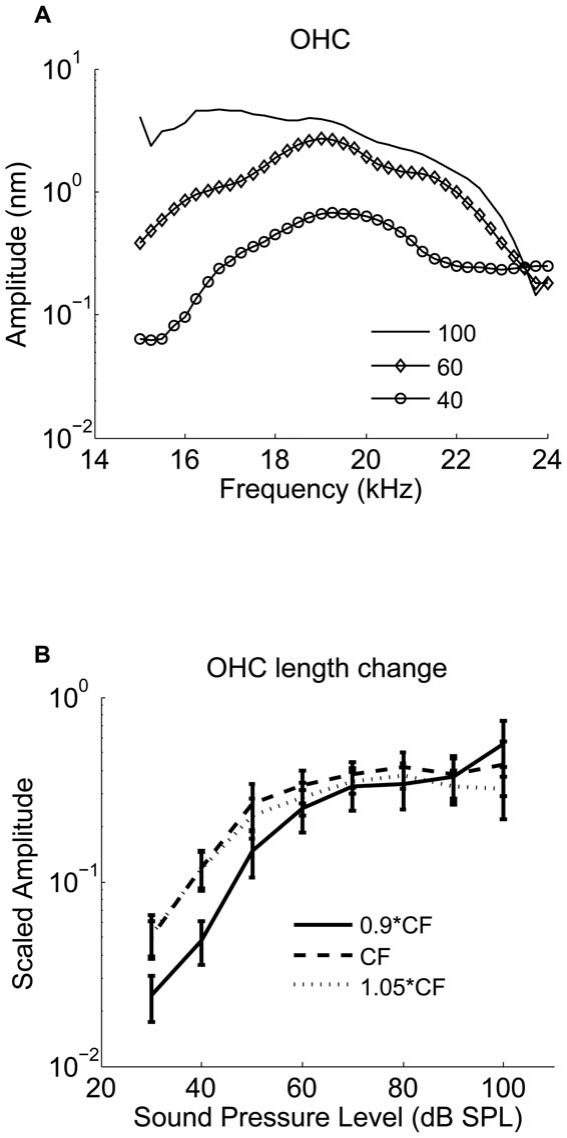
Outer Hair Cell length change (a) as a function of the frequency in a sensitive guinea pig cochlea (hearing loss<10 dB), (b) as a function of the sound level from the six sensitive animals.

**Figure 5:**
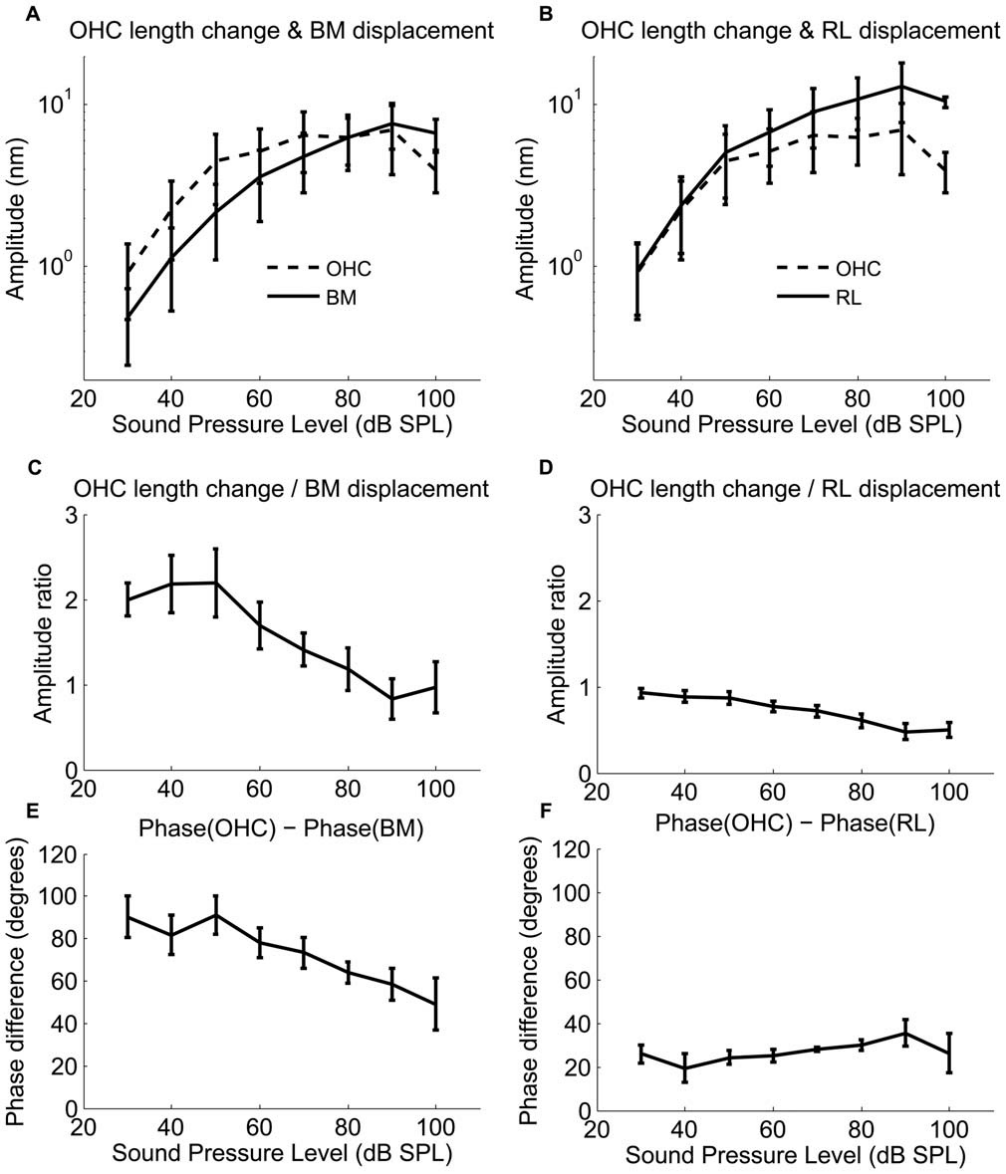
Outer hair cell length changes and phase differences between the OHCs length and the RL and the BM displacement as a function of the sound pressure level (dB SPL). **a**. Normalized relative OHCs length changes vs. BM displacement as a function of the sound pressure level between 30 and 100 dB SPL. **b**. Normalized relative OHCs length changes vs. the RL displacement as a function of the sound pressure level between 30 and 100 dB SPL. **c**. Phase difference between the OHC length changes and the BM displacements as a function of the sound pressure level between 30 and 100 dB SPL. **d**. Phase difference between the OHC length changes and the RL displacements as a function of the sound pressure level between 30 and 100 dB SPL. Data were obtained from six sensitive animals. Note the changes in the ordinate scales.

The displacement data can be used to obtain a measure of OHC length changes, since the Deiters' cells that connect the OHCs to the BM are very stiff and lack capacity for rapid movements [19], [20], [21]. The length change of the OHC depends on both stimulus intensity and frequency. At low levels, displacements peak near 20 kHz; the peak moves to lower frequencies as the stimulus level increases (**Fig. 4a**). This behavior is similar to the one observed at the RL. However, when plotting OHC length change at the best frequency as a function of stimulus level, a nonlinearity stronger than either the RL or BM becomes apparent (**Fig. 4b**, see also **Fig. 5a** and **b**). At and above the CF, OHC length changes saturate at around 70 dB SPL and then show a slight tendency to decline at the highest levels tested. The pronounced compression lends further support to the idea that OHC length change is a major factor in the nonlinearity of the cochlea [25].

### Visualizing the timing of OHCs length change within the organ of Corti

**Figure 6:**
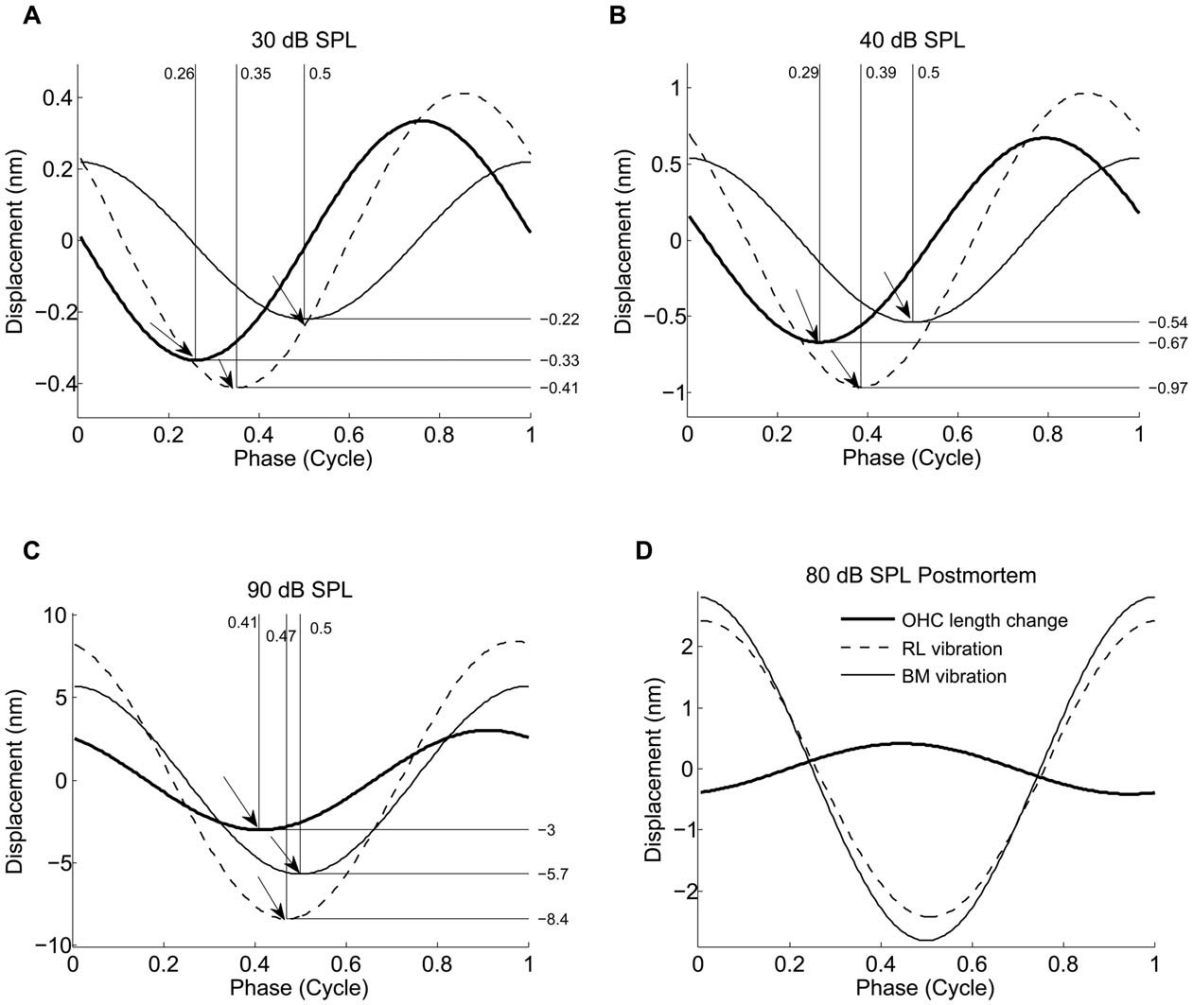
The timing of the BM and the RL displacement and the OHC contractile length change. The timing of the OHCs contraction and the RL and the BM displacement at the sound level of 30 dB (**a**), 40 dB (**b**) and 90 dB (**c**) from a sensitive animal (hearing loss<10 dB) and at the sound level of 80 dB from the postmortem animals. Positive direction is the direction to the SV for the RL and the BM displacements, and expansive for the OHC length-change. The arrow pointed at the OHC length change curve indicates the shortest position of the OHC, the arrow pointed at the RL vibration curve indicates the time (phase) when the RL has moved farthest into the ST, the arrow pointed at the BM vibration curve indicates the time (phase) when the BM has moved farthest into the ST. Note the phase difference between the OHC contractile length change and the RL and the BM displacement.

To illustrate the relationship between OHC length changes and other structures in the organ of Corti, the instantaneous values of RL, BM and OHC length changes are plotted as functions of time for BF tones at 30 dB SPL (**Fig. 6a**), 40 dB SPL (**Fig. 6b**), 90 dB SPL (**Fig. 6c**) for the sensitive animal and at 70 dB SPL for the postmortem animal (**Fig. 6d**). This information can also be equivalently represented by the relative phase (and magnitude) between the three structures at a given frequency. To distinguish the phase lead from the phase lag, it is important to note the wave sign convention. We use 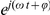 in our measurements and analysis, where positive ϕ is the phase lead. The RL and the BM displacements as a function of time over one cycle in **Fig. 6** are generated with the equation
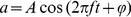
(1)The amplitude value *A* is given by the RL or BM value from one sensitive animal. For the RL, 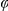 is the phase lead of RL displacement relative to the BM displacement (hence ϕ is zero for the BM; this analysis neglects possible radial motion components as these have previously been found to be small relative to the vertical motion [26]). OHC contraction is the difference between the BM and the RL displacements towards SV. The X-axis indicates one cycle. To be meaningful, data in this figure must be interpreted in the context of organ of Corti structure. The RL and the BM can be considered as approximately parallel beams (**Fig. 1**). As seen in **Figs. 2** and **3**, the RL displacements are larger than those of the BM. Consequently, the RL will approach the BM during motion directed at scala tympani, which implies OHC shortening (**Fig. 6a**, arrow pointed at the OHC length change curve, note that positive displacement in this figure means motion directed at the scala vestibuli for the BM and the RL and an elongation for the OHCs). The differences in phase mean that the OHCs are shortest when the BM moves with greatest velocity in the direction of the scala tympani (**Fig. 6a**). These phase relations depend on the stimulus level: phase differences decreases as the sound level increases, and at 90 dB SPL, the phase difference between the OHC contraction and the BM displacement decreases to 32° (**Fig. 6c**). The length changes also depend on the physiological state of the animal. Postmortem, the OHC length changes are small, and both the RL and the BM moved in phase (**Fig. 6d**).

These observations are inconsistent with the established theory and may therefore seem counter-intuitive. The geometric concept of organ of Corti mechanics is that the RL motion directed toward the scala vestibule causes excitatory deflection of the hair cell stereocilia [27]. The resultant hair cell contractile force would tend to draw the BM upward. Thus, in a similar way, established theory posits that the hair cell expansive force must be timed with the motion of the BM toward the scala tympani [14]. To interpret the OHC length changes, it is necessary to understand that the OHC length changes originate from forces produced by and/or acting upon these cells, especially at low stimulus levels. The phase relationship between the force generation and the length change is unknown and influenced by the mechanical impedance of the surrounding structures.

### OHC contraction relative to BM, RL displacement

To illustrate the relationship between the OHC length change and the displacement of the BM and the RL, we compare their absolute displacement magnitudes in **Fig. 5a** and **b**. Note that the OHC length changes are very similar in magnitude to the RL vibration at low levels, but show more pronounced saturation than either of the two structures. This behavior is also apparent in the normalized data presented in **Fig. 5c** and **d**. The normalized OHC length change is the ratio between the OHC length change and the BM or the RL displacement. **Fig. 5c** demonstrates that the OHC contraction relative to the BM displacement decreases with the sound level. The relationship is responsible for the apparent decrease in cochlear amplification gain that occurs with increasing sound level. The maximum OHC contraction is about two times the displacement of the BM at 30 dB SPL, decreasing to about 0.8 at 90 dB SPL. At 40 dB SPL, the maximum OHC contraction is 2.0 nm at 20 kHz (CF). **Fig. 5b** and **d** demonstrates that the OHC contraction relative to the RL decreases from 0.9 at 30 dB SPL to 0.4 at 90 dB SPL.

### Phase difference between OHC contraction and BM, RL displacement

To illustrate the timing relationship between the OHC contraction and the RL and the BM displacement, we plot the phase differences between the OHC length change and the RL and the BM displacement, as a function of sound pressure level from six sensitive animals. The OHC length changes do not occur in synchrony with the BM displacement but display a difference in phase that depends on the stimulus level (**Fig. 5c**, **d**).

In sensitive animals, the phase difference between the OHC contraction and the BM displacement to the ST is 0.24 cycles; the OHC contraction precedes the BM displacement to the ST, resulting in a relative phase lead of about 90° (**Fig. 6a** and **5e**). The phase systematically shifts as the sound level increases and at 90 dB SPL, the phase difference between the OHC contraction and the BM displacement to the ST is 0.09 cycles, which means only 32° of phase lead between the OHC contraction and the BM displacement remains (**Fig. 6c** and **5e**). The average phase difference between the OHC contraction and the BM displacement is 90±10° at 30 dB SPL, which is significantly different from zero, decreasing to 40±11° at 100 dB SPL (**Fig. 5e**). Postmortem, the phase and the amplitude of the two structures are the same (data not shown).

The phase difference between the OHC contraction and the RL displacement to the ST is 0.09 cycle at 30 dB SPL, the OHC contraction precedes the RL displacement, resulting in a relative phase lead of about 32° (**Figs. 6a** and **5f**). At 90 dB SPL, the phase difference between the OHC contraction and the RL displacement to the ST is 0.06 cycles, which means 22° of the phase lead between the OHC contraction and the RL displacement (**Fig. 6c**). The average phase difference between the OHC length change and the RL displacement was 26±4° at 30 dB SPL, and 26±9° at 100 dB SPL (**Fig. 5f**). Postmortem, the phase and the amplitude of the two structures are the same (data not shown). The 1∶1 ratio between the OHC length change and the RL displacement suggests that the OHCs control the displacement of the RL at low stimulus levels and that the BM makes a major contribution to the mechanical impedance of the cochlear partition, providing a scaffold for the OHCs. As the stimulus level increases, the organ of Corti vibrations are increasingly determined by the stiffness, mass, and friction inherent to the organ of Corti and the OHC length changes have a smaller role. This is to say that the power produced by OHC force acting over the OHC length change, becomes a proportionally smaller part of that needed to move the organ.

## Discussion

Experiments on isolated OHCs [12], [28] have demonstrated that the OHC contracts in response to depolarization and elongates when hyper-polarized. *In vivo*, alternating potentials have been measured both intracellularly [29] and in the extracellular space adjacent to the OHCs during sound stimulation [30]. These potentials could serve as a driving stimulus for OHC motility. *In vivo* (and *in situ*), the OHC vibration is constrained by the impedances of the BM, the RL and the TM. The extent to which the OHC vibration is constrained causes force to be applied on these structures. The total force is given by [31]:
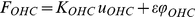
(2)The total force consists of the active force due to the OHC somatic electromotility [6], [7], [32], (second term in the right hand side of equation (2) and a passive force due to the OHC stiffness (first term in the right hand side of equation (2)). The force 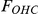 applied on the apical and basal ends of the OHC is contractile in equation (2) for a contractile displacement 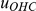 and depolarizing potential 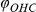.

It is important to distinguish between the length changes in the isolated OHCs *in vitro* versus the length changes measured *in vivo*. An isolated OHC; there are no forces acting on the OHC, thus 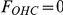. Therefore, from equation (2), the isolated OHC contraction is given by
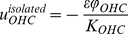
(3)where **ε** is the piezoelectric coefficient and a negative quantity. This relationship is consistent with the OHC contraction for depolarizing transmembrane potential.

Most researchers refer to the isolated OHC contraction 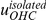 as the ‘length change’. The isolated cell length change is usually equated with the active force *in vivo*, and this force is given by
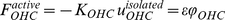
(4)In other words, the active force *in vivo* is directly associated with the electromotile length-change in isolated cells. The *in vivo* length change, though likely arising from the same process, is given by the OHC contraction 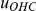 in equation (2). The *in vivo* OHC length change is an important aspect of the active process in the cochlea because the power transferred to the traveling wave, as proposed by cochlear models [33].

Our experimental data in this report demonstrates that the OHC length changes occur *in vivo* in the sensitive cochlea. The relative length changes decrease with the sound stimulus level and demonstrate compressive nonlinearity. These features are absent from the postmortem preparations, which lack amplification attributable to the OHCs. The maximum OHC contraction is about two times the displacement of the BM at 40 dB SPL, decreasing to about 0.8 at 90 dB SPL. The compression thus appears to be more pronounced in the OHC length-change compared to the BM and supports the hypothesis that the OHCs are the likely physiological site of the nonlinearity. Our experiments also show that the timing of length changes depends on the stimulus level. At low sound levels, the OHC begins to contract when the BM moves from scala vestibuli to the scala tympani; the OHC contraction phase leads the BM displacement to the scala tympani by about 90°.

In contrast to the living organ of Corti, the RL and the BM within the organ of Corti ,in the postmortem preparation, vibrate almost as a unit in response to the CF tones (**Fig. 2c**, **2d**, **Fig. 3b, 3d** and **Fig. 6d**), and there is no phase and amplitude difference between the RL and the BM. The disappearance of the amplitude and the phase difference between the BM and the RL postmortem certainly indicates a dependence on OHCs, as these cells are the only ones capable of changing cell length by generating force at the requisite speeds [8] and the effect of their activity is known to be highly dependent on the functional status of the cochlea [34]

In studies of excised cochleae [7], [35], electrical depolarization of the OHCs induces somatic contractions that cause the BM and the RL to be drawn together (**Fig. 1c, 1d**). These findings support [36] and form the basis of [37] the mechanical models of the BM tuning: with a phase lead of 90° between the OHCs contractile force and the displacement of the BM towards the SV, the OHCs would provide a properly timed force to enhance the BM's motion [5], [37], [38], [39]. An erroneous timing would result in decreased rather than increased hearing sensitivity. It is often assumed that the transduction current is maximal when the RL reaches its uppermost point of displacement; each OHC, therefore, is being depolarized, so it continues to shorten [40], [41]. Our data are not consistent with this idea, as the maximal OHC contraction occurs before the RL reaches its *lowermost* point (the OHC length changes have a phase lead over the displacement of the RL toward the scala tympani).

There are two primary reasons for these differences. Firstly, the phase of the OHC length change is not the same as that of the active force. Their phase difference is expected to be influenced by the mechanical impedances of the surrounding structures in the cochlear partition [42]. Secondly, many *in vitro* studies simply assume that the hair bundle deflection precedes the OHC length-change. If we assume that the hair bundle deflection is a simple function of the RL displacement, this would mean that the RL displacement should phase-lead the OHC length change. These studies have based their assumption on the open-loop system or experiments on isolated cells, where stereocilia deflection causes change in trans-membrane potential leading to the OHC length-change. *In vivo*, however, in addition to the hair bundle deflection causing the OHC length change, the bundle deflections could also result from the OHC length-change (which implies that the hair bundle deflection would lag the OHC length-change). In other words, the deflections *in vivo* are the result of a closed-loop feedback system, along with the intricate effect of the tectorial membrane and the traveling-wave [14], [43], [44], which is lacking *in vitro*.

The OHC electromotility is one of the two prime candidates for the active process leading to the amplification of the traveling wave in the cochlea. In isolated cells, the OHC electromotility process is known to cause length changes (contraction) in response to a change (depolarization) in the transmembrane potential. For the same process to transfer power to the traveling wave *in vivo* requires not only an active force, but also the affiliated active motion. For power addition to the traveling wave, it is usually said to require [14] that the active force have a component in-phase with the BM velocity. However, it is important to note that the power due to electromotility requires active level-dependent OHC length-changes in addition to the active OHC force. Although our analysis considers electromotility as the active process likely leading to the measured OHC length change *in vivo*, it remains open whether the length change we measured could also be influenced by the hair bundle motility [45], [46].

In conclusion, we show *in vivo* the OHCs length changes in the sensitive cochlea and that the length changes depend on the stimulus level. The level-dependent OHC length changes provide direct evidence of the active process *in vivo*, which is important for mammalian hearing sensitivity and frequency selectivity. Unless the OHCs contract and elongate *in vivo*, the active electromotile force cannot transfer the electromechanical power to the cochlear partition. Thus, being a key element in the active power transfer to the traveling wave, the OHC length changes demonstrated in this article provide vital information on the active process in the cochlea.

## Methods

### Optical coherence tomography

The vibration of the RL and the BM was measured with a low coherence interferometry (optical coherence tomography system) [22], [23]. The sample arm of this interferometer system is a microscopic system. Using a wide bandwidth light source of 1310±47 nm, the system achieves optical axis resolution of ∼7 um, without using the focus power of the objective lens, which is limited by the achievable numerical aperture of the optical system, determined by the access hole opened on the cochlear bony wall. The infra-red light also has penetration capability so that the vibration inside the organ at the RL can be measured.

### Experimental procedures

Albino guinea pigs (250–350 g) with normal hearing were used. All procedures in this study were reviewed and approved by the Institutional Animal Care and Use Committee at Oregon Health & Science University, approval date January 19, 2011 under Animal Welfare Assurance Number A3304-01. After anesthesia, the animal was placed in a customized head-holder and the cochlea surgically exposed. To measure the *in vivo* OHC length changes with the OCT system, it is necessary to have a good image of the organ of Corti to ensure that the measured points on the RL and the BM are at the appropriate radial coordinates. To obtain the image of organ of Corti, a sufficiently large opening (400–500 um diameter) was made in the otic capsule close to round window. The opening served to allow more focused light to fall on the organ and permitted observation across the entire width of the BM. CFs of the recording positions of the organ were 18.75–22.25 kHz and appropriate for the basal location of the opening near the round window.

During the experiment, a cross-section image was firstly acquired (as shown in **Fig. 1b**), guiding the selection of the measurement site. After that, the light was directed by two scanning mirrors to the selected site (RL or BM), and vibration measurement conducted with this homodyne interferometer system. The RL and BM selected locations were determined from the image by noting the shape of the group of three OHCs that lie at an angle to the optical axis. The RL location was in the center of the group while the BM location was at the location of an imaginary line passing through the long axis of the group of OHCs and intersecting the BM. Calibration was performed by two lock-in amplifiers (Stanford Research SR830) to decode the vibration amplitude and phase. Hearing sensitivity of the animal was monitored by recording the sound-induced auditory nerve response, via an electrode placed on the round window.

### Calculation of the OHC length change

In our OCT measurements, positive displacements are in the vertical direction and towards the scala vestibuli (SV). Therefore, the increase in the length of the OHC-DC complex is given by
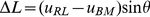
(5)Here θ is the angle made by the RL with vertical. Although the DC soma stiffness has not been directly measured, estimates based on the presence of the microtubules suggest a stiffness at least 1000 times higher than the OHC [19], [20], [21]. Based on this estimate, the increase in the length of the OHC is well approximated by the increase in the length of the OHC-DC complex given by 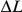.

The contractile displacement of the OHC is therefore given by
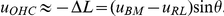
This OHC displacement is derived from the directly measured RL and BM displacements in sensitive guinea pigs *in vivo*. The angle θ is approximately 65°, as measured from our OCT images (**Fig. 1b**).
